# Editorial: The impact of alkalizing the acidic tumor microenvironment to improve efficacy of cancer treatment

**DOI:** 10.3389/fonc.2023.1223025

**Published:** 2023-05-31

**Authors:** Reo Hamaguchi, Shinji Uemoto, Hiromi Wada

**Affiliations:** ^1^ Clinical Cancer Research Team, Japanese Society on Inflammation and Metabolism in Cancer, Kyoto, Japan; ^2^ Department of Surgery, Shiga University of Medical Science, Otsu, Japan

**Keywords:** cancer, cancer metabolism, tumor microenvironment, alkalization therapy, multi-drug resistance, pH

In recent years, the acidic tumor microenvironment (TME) that is created by cancer-specific metabolism has attracted much attention in cancer therapy. In this Research Topic, we discuss the wide range of knowledge that has accumulated regarding cancer metabolism, focusing on the effects of acidity of the TME on cancer pathology. Points discussed include characteristics of the acidic TME (Bogdanov et al.), an overview of alkalization therapy (Hamaguchi et al., Wada et al.), a clinical trial of alkalizing agents on cancer patients (Gillies et al.), the association between acidic TME and glioblastoma (Seyfried et al.), the association between pH of the TME and the immunological state (Hosonuma and Yoshimura in press), role of the immunosuppressive TME in pancreatic cancer (Hashimoto et al.), *Drosophila* as an effective toolkit to investigate cancer metabolic abnormalities (Jiang et al.), acidic imaging positron emission tomography probes (^89^Zr-labeled pH-low insertion peptides) (Bauer et al.), role of the proton-sensing G protein-coupled receptor GPR68 in breast cancer (Elemam et al.), the association between cancer and chronic heart failure focusing on mitochondrial abnormalities (Takada et al.), and the association between cancer metabolism and ascorbic acid (Maekawa et al.). To address cancer metabolism and target it as a treatment, it is necessary to recognize how cancer develops, and what characteristics of the metabolic process are involved. Here, we will outline the origins and metabolism of cancer, how to deal with it, and the importance of alkalization of the TME.

How does cancer develop? The most important point was reported by Otto Warburg in “On the origin of cancer cells” (1). Cancer cells develop when there is a lack of oxygen, but a supply of nutrition. Cancer cells are primarily glycolytic, as they perform fermentation, meaning that they are dependent on the glycolytic system rather than oxidative phosphorylation by cellular respiration. The essence of this is the presence of mitochondria in eukaryotic cells. Cancers comprise cells that have been forced to choose their own path of life without working, but not failing, mitochondria. Gilles R. and Gatenby R. et al. reported in detail that cancer cells are dependent on aerobic glycolysis for survival as a result of Darwinian selection pressure (2). In addition, as Seyfried T. has stated, cancer can be considered as a metabolic disease (3). These points suggest that cancer cells try to survive on their own in an environment where there is a lack of oxygen but a supply of nutrients. In other words, cancer is comprised of cells that have lost their coordination with other cells in the body, and are living on their own.

How does cancer metabolism work? Cancer cells have a unique metabolism that differs from that of normal cells, and as enhanced glycolysis generates large amounts of acidic substances (protons) inside the cell, cancer cells expel protons to the outside of the cell by proton transporters, resulting in the inside of the cell being alkaline and the outside being acidic (4). The most important proton transporter that is involved in this phenomenon is sodium/proton (Na^+^/H^+^) exchanger isoform 1 (5). In the general biological environment, the extracellular pH of normal cells is maintained at pH 7.2 to 7.4, whereas the pH around cancer cells tends to be more acidic at pH 6.2 to 6.8 (6). This acidification of the TME has been reported to promote cancer progression. In this state, cancer cells become resistant to a variety of treatments, their proliferation is activated, and their metastatic potential is also increased (7, 8). In general, current cancer treatments do not target the pH balance of the TME that results from this cancer-specific metabolism. This means that adequate and satisfactory cancer treatment results have not yet been achieved.

What happens when the acidic TME is alkalized? Reversal of the pH gradient between the inside and the outside of cancer cells, i.e., extracellular acidification and intracellular alkalinization, attenuates the intracellular concentration of many anticancer drugs, and leads to resistance to anticancer drug treatments (5). For example, it has been reported that an increase in intracellular pH from 7.0 to 7.4, although in an experimental system, leads to a 2,000-fold increase in adriamycin resistance in human lung cancer cell lines (9). Conversely, lowering the intracellular pH (raising the extracellular pH) of cancer cells is expected to attenuate their resistance to various anticancer drugs, and to make anticancer drug therapy more effective. Furthermore, an acidic TME is known to decrease anticancer immune responses, and hence alkalinization of the acidic TME is expected to improve the function of immune cells, such as dendritic cells, natural killer cells, cytotoxic T cells, and macrophages (10, 11). In addition, this treatment method of lowering the intracellular pH (raising extracellular pH) may be sufficiently effective on its own (Wada et al.).

Clinical methods for alkalization of this acidic TME include alkalization therapy with alkalizing agents or proton pump inhibitors (Hamaguchi et al.). In addition, the influence of the daily diet should also be considered. Diets with alkalizing effects are rich in vegetables and fruits, which at the same time have anti-inflammatory and gut-regulating properties (12). Alkalization therapy is a treatment that acts on cancer metabolism, and can be used in combination with anticancer drugs, radiation therapy, and other therapies, and is also a safe treatment method. In the future, the combination of alkalization therapy and conventional therapy for the treatment of cancer needs to be further investigated in prospective clinical trials.

## Author contributions

RH and HW performed the literature review and wrote the article. SU performed the literature review. All authors conceived and designed the study and gave final approval for publication.
